# Genetic Variants and Tumor Immune Microenvironment: Clues for Targeted Therapies in Inflammatory Breast Cancer (IBC)

**DOI:** 10.3390/ijms22168924

**Published:** 2021-08-19

**Authors:** Yulan Gong, Rajeswari Nagarathinam, Maria F. Arisi, Lorenzo Gerratana, Jennifer S. Winn, Michael Slifker, Jianming Pei, Kathy Q. Cai, Zachary Hasse, Elias Obeid, Julio Noriega, Christopher Sebastiano, Eric Ross, Katherine Alpaugh, Massimo Cristofanilli, Sandra V. Fernandez

**Affiliations:** 1 Fox Chase Cancer Center, Philadelphia, PA 19111, USA; JenniferS.Winn@fccc.edu (J.S.W.); Michael.Slifker@fccc.edu (M.S.); Jianming.Pei@fccc.edu (J.P.); Qi.Cai@fccc.edu (K.Q.C.); Zachary.Hasse@fccc.edu (Z.H.); Elias.Obeid@fccc.edu (E.O.); Julio.Noriega@fccc.edu (J.N.); Eric.Ross@fccc.edu (E.R.); R.Alpaugh@fccc.edu (K.A.); 2 Department of Pathology, Thomas Jefferson University, Philadelphia, PA 19107, USA; Maria.Arisi@jefferson.edu (M.F.A.); c.sebastiano@healthnetworklabs.com (C.S.); 3 Feinberg School of Medicine, Northwestern University, Chicago, IL 60611, USA; Lorenzo.Gerratana@northwestern.edu (L.G.); Massimo.Cristofanilli@nm.org (M.C.)

**Keywords:** cell-free DNA (cfDNA), programmed cell death-ligand 1 (PD-L1), poly (ADP-ribose) polymerase, PARP inhibitor, immune checkpoint inhibitors (ICIs), tumor infiltrating lymphocytes (TILs)

## Abstract

To better understand the etiology of inflammatory breast cancer (IBC) and identify potential therapies, we studied genomic alterations in IBC patients. Targeted, next-generation sequencing (NGS) was performed on cell-free DNA (cfDNA) (*n* = 33) and paired DNA from tumor tissues (*n* = 29) from 32 IBC patients. We confirmed complementarity between cfDNA and tumor tissue genetic profiles. We found a high incidence of germline variants in IBC patients that could be associated with an increased risk of developing the disease. Furthermore, 31% of IBC patients showed deficiencies in the homologous recombination repair (HRR) pathway (BRCA1, BRCA2, PALB2, RAD51C, ATM, BARD1) making them sensitive to poly (ADP-ribose) polymerase (PARP) inhibitors. We also characterized the tumor-infiltrating lymphocytes (TILs) in tumor tissue biopsies by studying several markers (CD4, CD8, FoxP3, CD20, PD-1, and PD-L1) through immunohistochemistry (IHC) staining. In 7 of 24 (29%) patients, tumor biopsies were positive for PD-L1 and PD-1 expression on TILs, making them sensitive to PD-1/PD-L1 blocking therapies. Our results provide a rationale for considering PARP inhibitors and PD-1/PDL1 blocking immunotherapy in qualifying IBC patients.

## 1. Introduction

Inflammatory breast cancer (IBC) is a rare and aggressive form of breast cancer. Its diagnosis is based on inflammatory clinical signs (warmth, erythema, edema-peau d’orange) in more than 30% of the breast, with or without an underlying palpable mass [1,2,3]. Despite its name, IBC is not associated with a profuse inflammatory response; the characteristic redness and swelling of the breast are due to obstruction of lymphatic channels in the dermis by tumor cells [4,5]. In the United States (US), IBC accounts for 2–6% of all patients with breast cancer [6,7,8]. Although IBC is a relatively rare clinical subtype of locally advanced breast cancer, it is responsible for approximately 10% of breast cancer-associated deaths annually in the US, which translates into 4000 deaths per year [4,7]. Approximately 20–30% of IBC patients present with distant metastasis (stage IV disease) at diagnosis, compared to 6–10% of patients with non-inflammatory breast cancer (non-IBC) [9]. IBC is a heterogeneous disease and can occur as any of the five molecular subtypes, although it is most commonly either triple-negative (TN) or HER2 overexpressing [10]. TN breast cancer, which is defined by an absence of estrogen and progesterone receptors, and a lack of HER2 overexpression, has a worse prognosis than other subtypes [11]. The rarity of the disease, misdiagnosis, and difficulty in sample collection before treatment has resulted in a limited number of published molecular studies [2,12,13,14,15,16].

Data on risk factors for IBC are limited, and the contributions of hereditary versus environmental or lifestyle factors remain poorly understood [17]. Since IBC patients tend to be younger than other breast cancer patients, with a median age at diagnosis of 52 years compared to 57 for non-IBC patients, a genetic component is implied [18].

Although more recent data indicate an improvement in survival of IBC, the prognosis remains worse than non-IBC cases. The median overall survival (OS) for patients with stage III IBC is 4.75 years, compared to 13.40 years in those with non-IBC, and 2.27 years for stage IV disease in IBC versus 3.40 years in non-IBC patients [19,20]. The treatment of IBC remains similar to that of non-IBC and is primarily anthracycline/taxane-based neoadjuvant chemotherapy, and anti-HER2 agents for HER2+ tumors [21]. In order to develop targeted therapies specific to IBC, obtaining genomic information is crucial. Furthermore, studies on the IBC tumor microenvironment [13,14,22,23,24,25], including our previous studies [26], provide evidence for immune responses toward IBC, suggesting that patients may benefit from immune checkpoint inhibitors (ICI), such as PD-1/PD-L1 blocking antibodies.

To identify potential target therapies for IBC, we studied the somatic and germline variants via next-generation sequencing (NGS) performed on cell-free DNA (cfDNA) from blood (plasma or serum) and paired DNA from tumor tissue samples from 32 IBC patients. Whole blood was also studied in 20 of these patients to confirm germline variants. We recently showed high concordance between variants detected in tumor tissue and paired cfDNA when both samples were collected at the same time point [27]. Furthermore, we characterized the tumor-infiltrating lymphocytes (TILs: T-lymphocytes, B-lymphocytes, and macrophages) in tumor tissue biopsies from 24 IBC patients by studying markers of the immune response.

## 2. Results

### 2.1. Patient Characteristics and Samples Studied by Next Generation Sequencing (NGS)

Genetic variants were studied in 32 patients with advanced clinical stage III (7 patients) or IV (25 patients) IBC. All the patients were female, 25 were White (1 Hispanic, 1 Ashkenazi Jewish), 5 African-American, and 2 were Asian (Table 1). The median age at IBC diagnosis was 47.5 years (range 32–69) with 4 patients being ≥61 years old (Table 1).

At the time of diagnosis, the disease was classified as triple negative (TN) in eighteen patients (56.3%); nine patients (28.1%) had ER+ Her2− IBC disease; three patients (9.4%) had ER+ Her2+ IBC; and two patients (6.3%) had ER− Her2+ IBC (Table 1). Changes in ER or Her2 receptor expression were seen in five patients on subsequent sampling (Pt-12, Pt-20, Pt-28, Pt-29, and Pt-30) (Table 1).

Several other potentially relevant clinical factors are worth noting. Six of the patients (6/32; 18.8%) were initially diagnosed and treated for non-IBC breast cancer in the ipsilateral breast before being diagnosed with IBC (Pt-1, Pt-6, Pt-11, Pt-18, Pt-19, and Pt-25). With regard to reproductive history, 20 patients were parous at the time of disease onset (20/29; 69%) and 9 patients were nulliparous (9/29; 31%) (Note that the population N for this group is lower than the overall total because pregnancy data was not recorded for three patients). Concomitant genetic or autoimmune diseases were reported in 6 of the 32 total patients including Ehlers-Danlos syndrome, Factor V Leiden, Hashimoto’s Disease, Sjogren’s syndrome, inflammatory bowel disease, Graves’ disease, and rheumatoid arthritis. Of the 32 IBC patients, 25 continued their clinical follow-up at the Fox Chase Cancer Center (FCCC) and data regarding their disease progressions was available. Of these 25 patients, 6 (24%) developed brain metastases (Pt-1, Pt-2, Pt-15, Pt-27, Pt-29, and Pt-32) and two (Pt-15 and Pt-27) also had leptomeningeal disease. IBC patients (18/25) also developed metastasis to the lung, bone, liver, and/or abdomen.

Genetic variants were studied by NGS in malignant tissue or cells from malignant pleural effusions and paired cfDNA from peripheral blood (plasma or serum) in 32 patients. A total of 82 samples were studied: skin biopsies from 14 patients, breast tissue biopsies from 6 patients, a lymph node from 1 patient, cells from malignant pleural effusions from 7 patients, ascites from 1 patient, 33 samples of cfDNA (plasma or serum), and 20 samples of normal tissue or whole blood (Tables S1–S3). A panel of 93 breast cancer genes (Table S4) was used for targeted NGS, and variant call format (VCF) files were generated using the Qiagen GeneGlobe portal.

### 2.2. Clinical Relevant Variants in IBC Patients

**Figure 1:**
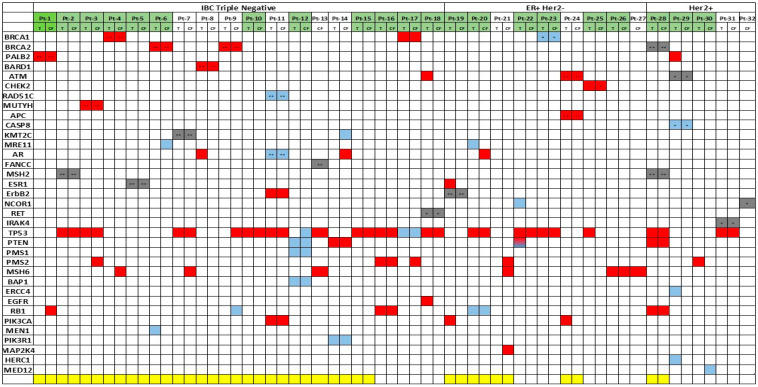
Genetic variants in tumor tissue and paired cfDNA in IBC patients. Germline and somatic variants detected in tumor tissue (T) and/or cfDNA (CF) are indicated for each patient. Pathogenic, likely pathogenic, and variants of uncertain significance are shown in red, blue, and grey, respectively. Samples in which T and CF were collected at the same time point are indicated in green. In yellow, patients in which normal tissue or whole blood samples were also studied by NGS to confirm germline variants. ** Confirmed germline variants. * Putative germline variants. For Pt-1, cfDNAs from month 12 (cfDNA1) and month 22 (cfDNA2) were studied, and the *PALB2* variant was detected in both, but the *RB1* mutation was only detected in CF from month 22.

**Figure 2:**
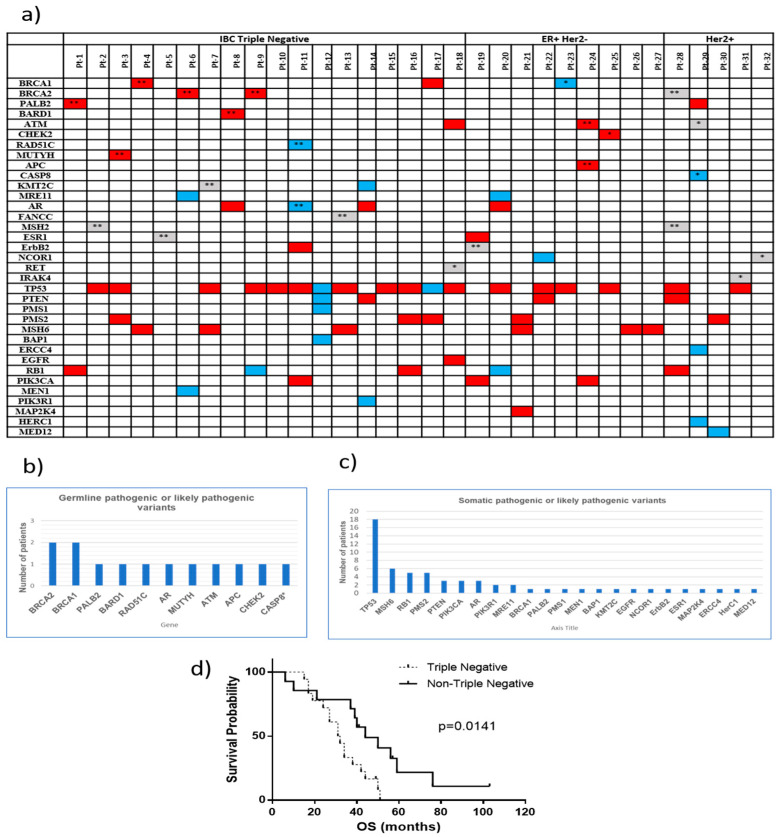
Genetic variants in IBC. (**a**) Germline and somatic variants detected in each patient. Pathogenic, likely pathogenic, and variants of uncertain significance, in red, blue, and grey, respectively. ** Confirmed germline variants by NGS of normal tissue or whole blood. * Putative germline variants; (**b**) Germline pathogenic and likely pathogenic variants; (**c**) Somatic pathogenic or likely pathogenic variants; (**d**) Kaplan–Meier curves for triple negative (TN) and non-TN. The median OS is 31.5 and 44 months for TN and non-TN, respectively.

The QIAGEN Clinical Insight Interpreter (QCI) (Qiagen, Redwood City, CA, USA) was used to classify the clinical relevance of coding variants in all the samples from the 32 IBC patients. In Figure 1, pathogenic, likely pathogenic, and variants of uncertain significance detected in tumor tissue and/or paired cfDNA (plasma or serum) for each patient are shown (*n* = 61 samples). For 29 of the 32 patients, variants were studied in tumor tissue and cfDNA; and for 3 patients (Pt-13, Pt-27, and Pt-32), these studies were performed using only cfDNA since tumor tissues were not available (Figure 1). Germline variants are those found in both cfDNA (from plasma or serum) and paired tumor tissue at an allele frequency (AF) ~50% or ~100%; their presence in normal tissue or whole blood (white blood cells) confirm their germline origin. Control samples such as normal tissue or whole blood were available for 20 patients (Pt-1 to Pt-15, Pt-19, Pt-20, Pt-21, Pt-24, and Pt-28) to perform validation of germline variants by NGS studies (Figure 1). To simplify, Figure 2a shows clinically relevant variants in each patient without indicating the source of the sample.

Of the 32 total patients whose samples were analyzed, 11 (34.4%) carried at least one pathogenic or likely pathogenic germline variant (Figure 2a). Seven of the 18 patients with TN (38.9%) and 4 of the 14 patients with non-TN (28.6%) disease carried at least one pathogenic or likely pathogenic germline variant. Germline pathogenic or likely pathogenic variants were observed in *BRCA2* (2/32), *BRCA1* (2/32), *PALB2* (1/32), *BARD1* (1/32), *RAD51C* (1/32), *AR* (1/32), *MUTYH* (1/32), *ATM* (1/32), *APC* (1/32), *CHEK2* (1/32), and *CASP8* (1/32) (Figure 2b). All these variants were carried at ~50% AF (mono-allelic). Germline variants in *ATM*, *BRCA1*, *BRCA2*, *CHEK2*, and *PALB2* were associated with a high risk of breast cancer; a more modest risk was associated with *BARD1* and *RAD51C* [28]. Monoallelic germline pathogenic mutations in *MUTYH* are associated with a moderately increased risk of colorectal cancer [29], and although their relation with breast cancer risk is controversial, some studies had reported an increased risk [29,30,31]. Most of the patients of our cohort had a family history of cancer (29/32) and seventeen of the patients (53.1%) had a family history of breast cancer in particular. Of those patients, 7 had a family history of breast cancer at a young age (<47 years old). Other cancers in their families included prostate (28.1%), colon (25%), and skin (15%) cancers. Seven patients also had a family history of gynecological cancers such as ovarian, cervical, endometrial, uterine, or fallopian tube cancers. A description of the changes in DNA and protein sequences for germline variants found in IBC patients is shown in Table 1.

Somatic pathogenic or likely pathogenic variants were detected mainly in *TP53* (18/32; 56.3%), *MSH6* (6/32; 18.8%), *PMS2* (5/32; 15.6%), and *RB1* (5/32; 15.6%); others somatic mutations were seen in *BRCA1*, *PALB2*, and *MRE11* (Figure 2c). DNA and protein changes for each variant are indicated in Tables S1–S3.

A statistically significant difference in OS was observed between the TN and non-TN groups, with the median OS at 31.5 months for patients with TN IBC and at 44 months for patients with non-TN IBC (Figure 2d).

Variants were also classified as biomarkers for a clinically available intervention (Tiers 1–3) (Tables S1–S3). Pathogenic germline *BRCA1* (Pt-4) and *BRCA2* (Pt-6, Pt-9) variants are classified as T1A since there are two poly (ADP-ribose) polymerase (PARP) inhibitors that have been approved by the FDA for patients with metastatic breast cancer who carry germline mutations in these genes [32,33]; olaparib and talazoparib inhibit the PARP enzyme, which is involved in DNA repair. In addition, PARP inhibitors are being tested in clinical trials for patients with somatic mutations in BRCA1/2 (ClinicalTrials.gov Identifier: NCT03286842), and mutations in other genes involved in DNA homologous recombination repair (HRR) such as *PALB2*, *BARD1, ATM, CHEK2*, and *RAD51C* (ClinicalTrials.gov Identifier: NCT02401347). In the cohort studied, 12 patients had tumors with pathogenic or likely pathogenic variants in those HRR genes being germline (Pt-1, Pt-4, Pt-6, Pt-8, Pt-9, Pt-24, Pt-25) or somatic (Pt17, Pt-18, Pt-29).

These results show that 31% of the IBC patients from this cohort carried pathogenic or likely pathogenic variants in genes involved in DNA homologous recombination repair (HRR) making them susceptible to PARP-inhibitors.

### 2.3. Concordance of Variants in Tumor Tissue and Paired cfDNA

**Figure 3:**
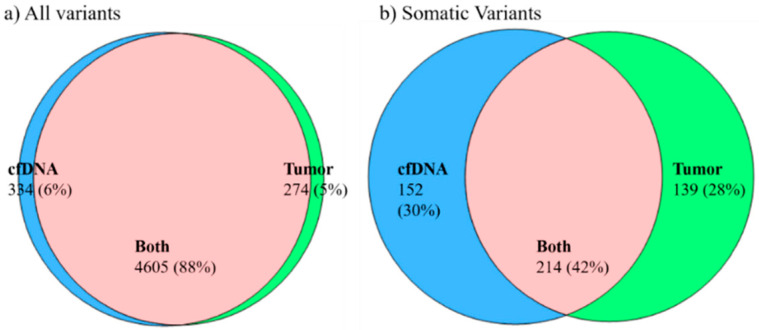
Variant concordance. Concordance between variants in tumor tissue and paired cfDNA.

We studied concordance between variants detected in tumor tissue and paired cfDNA. We included only those patients in which tissue and cfDNA were collected at the same time point, and normal tissue or whole blood were available. We considered concordant alterations only when the exact same sequencing alteration was present in both malignant tissue/cells and paired cfDNA. Variants causing coding changes of uncertain significance, and those pathogenic, likely pathogenic, benign, and likely benign were considered in studying concordance between tumor tissue and paired cfDNA samples. A total of 33 samples from 11 patients (tumor tissue/cfDNA/normal tissue) were included in this analysis: Pt-1 to Pt-5, Pt10, Pt-12, Pt-15, Pt-19, Pt-20, Pt-28. In these patients, 88% of all variants (germline and somatic) were detected in both tumor and paired cfDNA; 6% were only detected in cfDNA and 5% of the variants were only detected in tumor tissue samples (Figure 3a). To study concordance only for somatic variants, variants detected in normal tissue (or whole blood) were filtered out from variants detected in tumor tissue and cfDNA samples for each patient. In these patients, 42% of somatic variants were detected in both tumor tissue and paired cfDNA, 30% of somatic variants were detected only in cfDNA, and 28% somatic variants were detected only in tumor tissue samples (Figure 3b).

These results indicate that the genetic profiles from cfDNA and tumor tissue samples are complementary. If tumor tissue is not available, cfDNA from plasma or serum can be used to study the genetic profile in Stage III and IV IBC.

### 2.4. PD-1 and PD-L1 Expression in Tumor Biopsies

Our previous study showed that some IBC patients expressed PD-L1 on tumor biopsies suggesting that they could benefit from PD-1/PD-L1 immunotherapies [26]. Here, we evaluated PD-1 and PD-L1 expression by IHC in tumor biopsies in a larger cohort of 24 IBC patients. The anti-PD-L1, clone SP142, and anti-PD-1 clone NAT105 were used, and PD-L1 and PD-1 were evaluated as the percentage area of positive staining within tumor-infiltrating lymphocytes (TILs) (Table 2). Cases with PD-L1 < 1% were considered negative and ≥1% were considered positive (Table 2). A total of 7 of 24 IBC patients (29%) showed both PD-L1 and PD-1 expressions in tumor biopsies, and 17 patients had PD-L1 and PD-1 negative tumors (Table 2).

**Figure 4:**
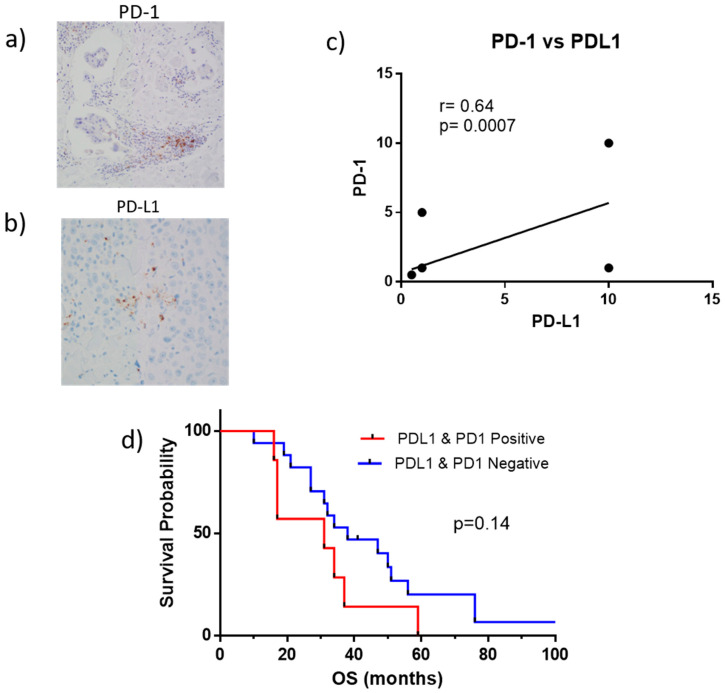
PD-1 and PD-L1 expression in IBC tumor biopsies. (**a**) Representative IHC staining of PD-1 (200× magnification); (**b**) representative staining for PD-L1 (200× magnification); (**c**) correlation between PD-1 and PD-L1 using a Spearman test; (**d**) Kaplan–Meier survival curves for IBC patients with PD-L1 and PD-1 positive (Median OS = 31 months) and PD-L1 and PD-1 negative tumors (median OS = 38 months).

From the 7 patients with PD-L1 and PD-1 positive biopsies, 4 (57%) had TN disease (Pt-3, Pt-5, Pt-14, and Pt-15) and 3 ER− Her2+ IBC (Pt-31, Pt-32 and Pt-33) (Table 2). Biopsy details are provided in Table S5. Representative IHCs are shown in Figure 4. PD-1 expression was only observed on tumor-infiltrating immune cells, and while PD-L1 expression may be observed on a variety of cell types, it was observed exclusively on TILS, which were morphologically identified by three qualified certified pathologists (YG, RN, CS) (Figure 4a,b). A positive correlation was observed between the co-expression of PD-1 and PD-L1 (Figure 4c). Patients with PD-L1 and PD-1 positive tumors had a lower OS than patients with PD-L1 and PD-1 negative tumors, though the difference did not reach statistical significance (*p* = 0.14) (Figure 4d). The median OS for PD-L1 and PD-1 positive tumors was 31 months, and for PD-L1 and PD-1 negative tumors was 38 months (Figure 4d).

Our results indicate that 29% of IBC patients in this cohort expressed both PD-L1 and PD-1 in their tumors and could benefit from PD-1/PD-L1 immunotherapies.

### 2.5. Correlation of Tumor Infiltrated Lymphocytes (TILs), PD-1 and PD-L1 in Tumor Biopsies

**Figure 5:**
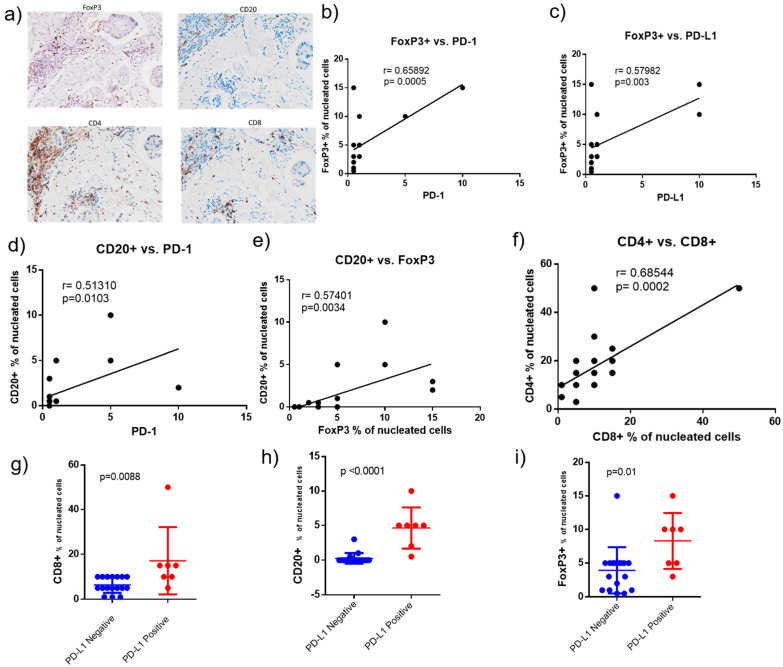
Tumor infiltrated lymphocytes in IBC. (**a**) Representative IHC for FoxP3 (Tregs), CD20+ (B cells), CD4+ (helper T cells) and CD8+ (cytotoxic T cells) cells are shown (200× magnification). Correlation between markers was studied using the Spearman test. Correlation is shown for: (**b**) FoxP3+ cells and PD-1; (**c**) FoxP3+ cells and PD-L1; (**d**) CD20+ cells and PD-1; (**e**) CD20+ cells and FoxP3; (**f**) CD4+ and CD8+ cells. The percentage of different immune cells in PD-L1 negative and PD-L1 positive tumors are shown: (**g**) Percentage of CD8+ cells (cytotoxic T cells); (**h**) percentage of CD20+ cells (B-cells); (**i**) percentage of FoxP3+ (Tregs) cells.

We assessed whether PD-1 and PD-L1 in the tumor samples correlated with TILs. The tumors were stained by IHC for FoxP3 to quantify regulatory T cells (Treg), CD20 to quantify B cells, CD4 to quantify helper T cells, CD8 to quantify cytotoxic T cells, and CD68 to quantify macrophages (Table 2). The infiltrating immune cells were quantified as the percentage of positive cells relative to the nucleated cells within each tumor and immediate peri-tumoral space. Quantifications were performed by three qualified pathologists (YG, RN, KQC). Representative IHC stains for FoxP3, CD20+, CD4+, and CD8+ cells are shown in Figure 5a.

Positive correlations were observed between FoxP3 (Treg) and PD-1 (Figure 5b); FoxP3 and PD-L1 (Figure 5c); CD20+ (B cells) and PD-1 (Figure 5d); and CD20+ and FoxP3+ cells (Figure 5e). There was also a positive correlation between CD4+ and CD8+ cells (Figure 5f).

Our results show that PD-L1 positive tumor biopsies had significantly more CD8+ (cytotoxic T-cells), CD20+ (B-cells), and FoxP3+ (Tregs) cells than PD-L1 negative tumors (Figure 5g–i).

## 3. Discussion

NGS technology is increasingly being used to study genetic variants in liquid biopsies, mainly cfDNA from plasma. An advantage of using liquid biopsies is that blood cfDNA provides molecular information about primary tumors as well as metastases and can be used to follow response to treatment. Recently, we showed that the genetic profile measured in blood cfDNA is complementary to that of tumor tissue [27]. In the present work, 88% of all variants (germline and somatic) were found in tumor tissue and paired blood, and 6% of all variants were detected only in cfDNA and 5% only in tumor tissue samples. Regarding somatic variants, 42% were detected in both cfDNA and paired tumor tissue samples, and 30% of somatic variants were detected only in cfDNA and 28% only in tumor tissue samples. These results confirm that the genetic profile of cfDNA and tumor tissue are complementary, and blood cfDNA can be used if tissue is not available or to follow variants along with treatment. Further studies are ongoing to improve the sensitivity of cfDNA using deeper sequencing, and larger panels to study additional cancer-related genes.

Most germline and somatic pathogenic or likely pathogenic variants found in IBC patients in this study correspond to proteins involved in DNA damage repair (BRCA1, BARD1, BRCA2, PALB2, RAD51C, MUTYH, ATM, PMS1, PMS2, MSH2, MSH6), cell cycle control (RB1, TP53, CHEK2) and apoptosis (APC, CASP8). This is in support of the literature which shows that DNA repair is more frequently altered in IBC than non-IBC (33% vs. 17%) [13]. Our study has shown that ~34% of patients carried at least one germline pathogenic or likely pathogenic variant, and this was more commonly found in the TN subtype (38.9%) than in non-TN IBC (28.6%). Though the frequency of patients carrying pathogenic or likely pathogenic germline variants is higher than the 14.4% reported in a recent study [34], these differences may be because our cohort included patients diagnosed at an older age and patients with Her2+ disease. These patient characteristics do not meet the genetic testing criteria under the current guidelines, which recommend testing for women with IBC who also have a family history of breast or ovarian cancers [35]. Here, we found that ~53% of IBC patients had a family history of other cancers such as prostate, colon, and skin cancers. Based on our data, it seems that germline mutation testing for all women diagnosed with IBC could provide important information impacting their individual treatment decisions as well as screening recommendations for their family members.

Since our study showed that 31% of IBC patients had somatic and/or germline (pathogenic or likely pathogenic) mutations in genes involved in the homologous recombination repair (HRR) pathway (*BRCA1*, *BRCA2*, *PALB2*, *RAD51C*, *BARD1*, *ATM*), these patients would benefit from PARP inhibitors. While patients with germline and somatic *BRCA1* or *BRCA2* mutations are particularly sensitive to PARP inhibitors, a substantial number of patients who lack these mutations still have tumors that are susceptible to PARP inhibitors [36]. Deficiencies in RAD51, PALB2, ATM, and CHEK2 were demonstrated to be associated with PARP inhibitor sensitivity [37,38]. Two PARP inhibitors, olaparib and talazoparib, have been approved by the FDA for women with metastatic breast cancer who carry germline mutations in *BRCA1* and *BRCA2* [32,33]. PARP inhibitors are being tested in clinical trials for patients with somatic mutations in BRCA1/2 (ClinicalTrials.gov Identifier: NCT03286842), and mutations in other genes involved in HRR such as *PALB2*, *BARD1, ATM, CHEK2*, and *RAD51C* (ClinicalTrials.gov Identifier: NCT02401347). Reversion mutations in HRR genes such as *BRCA1*, *BRCA2*, *RAD51C*, *RAD51D*, and *PALB2* have been observed in ovarian cancer patients who developed PARP inhibitor resistance [39], and the genomic analysis of sequential cfDNA from plasma samples might allow clinicians to follow these genes along with treatment in order to anticipate which patients might have developed resistance.

Tumor cells with HRR-deficiency can repair DNA by non-homologous end joining (NHEJ) DNA repair mechanisms, leading to the accumulation of somatic mutations that result in neoantigen formation and activation of the immune system. Immune responses seem to be mediated by CD8+ T lymphocytes within the tumor, although additional cell types (such as CD4+ T cells) may also contribute to immune surveillance [40,41]. Recruited Tregs promoted tumor survival by maintaining CD8+ cytotoxic T cell exhaustion and thereby preventing tumor cell clearance [42]. Programmed cell death ligand 1 (PD-L1) on tumor and/or immune infiltrating cells is a key immune checkpoint molecule that interacts with its receptor, programmed cell death protein 1 (PD-1) from T cells. PD-L1 and PD-1 interaction suppresses T cell-mediated immune responses and plays a role in immune escape by tumors [43].

In this study, as many as 29% of patients expressed PD1-L1 and PD-1 in tumor-infiltrating lymphocytes (TILs), and therefore had responded to the tumor at some stage of cancer development. PD-L1 expression correlated with PD-1 expression, as we previously observed [26]. We found that PD-L1 and PD-1 positive tumors had more CD8+ (cytotoxic T cells), CD20+ (B-lymphocytes), and FoxP3+ (Tregs) cells than PD-L1 and PD-1 negative IBC tumors. We also found that there was a positive correlation between PD-1 and B cells. Two groups recently found that expression of PD-L1 in IBC tumor samples correlated with higher stromal TILs that were highly enriched in B cells [22,25]. Immune checkpoint inhibitors (ICIs), which block PD-1 and PD-L1 interaction, play a role in the reversal of T cell exhaustion and clinical data suggests that patients whose tumor overexpress PD-L1 exhibit better clinical response to immune checkpoint therapy [44]. Furthermore, patients with a pre-existing T-cell-infiltrated tumor microenvironment are more likely to respond to immune-checkpoint inhibition [45].

Since it has been shown that PARP inhibitors upregulate PD-L1 expression in breast cancer cell lines [46], the combination of PARP inhibitors with anti-PD-L1 therapy could significantly increase therapeutic efficacy in those IBC patients with HRR mutations. Indeed, a recent study demonstrated that PARP inhibitors combined with immunotherapy can improve therapeutic effects [47]. The MEDIOLA study, a phase II clinical trial that explores the effective combination of Olaparib (PARP inhibitor) and durvalumab (anti-PD-L1 mAb) in the treatment of advanced solid tumors including TN breast cancer has already delivered encouraging results [48]. Thus, combining PARP inhibitors with PD-L1/PD-1 blocking therapies may be of special interest in IBC patients that harbored tumors with HRR mutated genes.

In conclusion, our results show a high incidence of germline variants in patients with IBC, supporting the idea that genetic factors play an important role in the development of IBC. This data, if validated in a larger cohort, would demonstrate the power that genomic analysis has to identify women at a high risk of developing IBC who would benefit from early screening. By studying genetic variants and the tumor immune microenvironment in IBC patients, our study suggests that IBC patients with HRR mutations could have benefited from the combination of PARP-inhibitor and PD-L1/PD-1 blocking therapies. Meanwhile, patients that only show PD-L1 expression would benefit from PD-L1/PD-1 inhibitors.

## 4. Materials and Methods

### 4.1. Patient Cohort

The samples used in this study were from IBC patients who were treated at Fox Chase Cancer Center (FCCC) between 2011–2016. The ethics approvals of this study were granted by the institutional review board (IRB) at FCCC as protocols 21-9024 and 16-9043. Patients signed an informed consent and HIPAA certification from the Human Subject Protection Committee prior to sample collection. Retrospective chart reviews were performed in order to collect data including age at diagnosis, family history of cancer, reproductive history, other diseases, and hormone receptor subtype.

### 4.2. Sample Collection for NGS

For serum isolation, blood was allowed to clot for 30–60 min and centrifuged at 2000× *g* for 20 min. The serum was then separated and stored at −80 °C. To isolate plasma, blood was placed into acid citrate dextrose (ACD) tubes and centrifuged for 15 min at 2000× *g*. The plasma was then separated and stored at −80 °C for further cfDNA isolation. For some patients, tumor tissue biopsies or cells from malignant pleural effusions were preserved in optimal cutting temperature compound (OCT) at −80 °C, while formalin-fixed paraffin-embedded (FFPE) blocks were prepared for others. Cells from malignant pleural effusions and ascites fluids were centrifuged at 1000× *g* for 10 min to create a cell pellet. The pellets were then suspended in 0.2% NaCl and an equal volume of 1.6% NaCl was added to induce red blood cell (RBC) hemolysis. This mixture was centrifuged again at 1000× *g* for 10 min, and the hemolysis step was repeated to remove all the remaining RBCs. Finally, the cell pellets were washed with phosphate-buffered saline, and the cell pellets were preserved in OCT at −80 °C or used to prepare FFPE blocks.

### 4.3. cfDNA and Genomic DNA Isolation

For the plasma and serum samples, ~10 mL was used to isolate cfDNA. Samples were centrifuged at 16,000× *g* for 10 min and the QIAamp MinElute ccfDNA Midi Kit (Qiagen) was used for cfDNA isolation. For DNA isolation from whole blood to study germline variants, the QIAamp DSP DNA Blood Mini Kit (Qiagen, Redwood City, CA, USA) was used. Genomic DNA was isolated from either the OCT-preserved or from FFPE blocks from malignant pleural effusion cells or tumor tissues biopsies. Ten 5 µm unstained sections from FFPE blocks were used for DNA isolation. Genomic DNA from frozen samples was isolated using the QIAamp DNA Micro Kit (Qiagen, Redwood City, CA, USA), and the GeneRead DNA FFPE Kit (Qiagen, Redwood City, CA, USA) for FFPE sections. The isolated DNA samples were quantified using a Qubit fluorometer and then used to prepare libraries for targeted NGS.

### 4.4. Library Preparation for NGS

Libraries were prepared using the QIAseq Targeted DNA Panel, Human Breast Cancer Panel DHS-001Z (Qiagen, Redwood City, CA, USA), and the QIAseq 12-index (Qiagen, Redwood City, CA, USA). The DHS-001Z breast cancer panel covers 93 genes (Table S4) and contains 4831 primers. It is able to detect both single nucleotide variants (SNVs) and small indels (insertions and deletions); copy number variants were not studied. cfDNA from plasma or serum (~45 ng) and genomic DNA from tumor tissue or cells (~200 ng) were used for NGS-library preparation (Table S6). Seraseq ctDNA reference material v2 of AF 2% (Cat# 0710-0203, SeraCare, Milford, MA, USA) and wild-type Seraseq ctDNA reference material v2 WT (Cat # 0710-0208, SeraCare, Milford, MA, USA) were used to prepare controls for allele fractions 2%, 1%, and 0.5%. These ctDNA controls were run in order to establish the ability to detect the pre-established variants and to determine the lower limit of detection of our assays. Whole blood or normal tissue from FFPE samples were available for 20 IBC patients (Pts 1–15, 9, 20, 21, 24, and 28) to perform validation of germline variants.

### 4.5. NGS and Data Analysis

Library preparation included sequence barcodes to discriminate samples and unique molecular indices (UMIs) to identify PCR duplication. Four libraries were pooled together per flow cell and sequenced (Illumina MiniSeq with high-output kits) producing an average of 6,923,890 total reads per sample and 2270 paired-end reads per targeted region (Table S6). The data was analyzed using Qiagen’s GeneGlobe portal and the variant call format (VCF) files were generated. Variants were filtered out if no allele fraction (AF) was greater than 2% for tumor tissue samples or malignant cells, and greater than 2% for blood cfDNA. AF for a specific variant can be defined as the number of times that variant is observed divided by the total number of reads of that region. In case whole blood or normal tissue was available, germline variants were validated. A total of 82 patient samples were studied by NGS: 33 blood cfDNA samples from 32 patients (18 plasma and 15 serum samples); 29 paired tumor samples from 29 patients (14 skin biopsies, 6 breast biopsies, 7 samples of tumor cells from malignant pleural effusions, 1 sample of malignant ascites and 1 lymph node); and DNA isolated from whole blood or normal tissue from 20 patients to confirm germline variants. Putative germline variants were considered those with an AF around 0.5 (or 50%) or 1.0 (or 100%) in blood cfDNA and tumor tissue (or cells from malignant pleural effusions).

### 4.6. Variant Classification

Qiagen Clinical Insight Interpret (QCI Interpret, Qiagen, Redwood City, CA, USA) was used to annotate variants. QCI Interpret evaluates variants by matching to a database of published supporting evidence and returns classifications using consensus guidelines for variants predicted to be pathogenic or likely pathogenic, benign or likely benign, or of uncertain significance [49,50]. It also classifies variants according to its clinical actionability; clinical actionability subcategories are provided based on levels of evidence according to the guidelines [49,50]. Tier 1 indicates strong clinical significance, with level A variants to predict response or resistance to therapies approved by the FDA for specific types of tumors (in this case, breast cancer), and level B variants predicted to affect therapy based on well-powered studies or smaller studies that are confirmed or reproduced by different groups [49,50]. Tier 2 indicates potential clinical significance, and includes level C which indicates evidence of an effect on FDA-approved therapies for different tumor types or investigational therapies (Tier 2C: off-label treatments), while Tier 2D variants are supported by evidence from preclinical trials or a few case reports [49,50].

### 4.7. Concordance of Genetic Variants in Tumor Tissue and Paired cfDNA from Plasma/Serum

A total of 33 samples from 11 patients (tumor tissue/cfDNA/normal tissue) were included in this analysis: Pt-1 to Pt-5, Pt10, Pt-12, Pt-15, Pt-19, Pt-20, Pt-28. We included only those patients in which tissue and cfDNA were collected at the same time point, and if normal tissue or whole blood were available. Concordance was defined as detecting an identical sequencing variant in tumor tissue (or cells from malignant pleural effusions) and paired cfDNA from plasma or serum. Variants with allele frequency lower than 2% were filtered out. Exonic and/or splicing variants were retained; intronic variants were filtered out. Variants of uncertain significance, and those pathogenic, likely pathogenic, benign, and likely benign were used for the concordance calculations.

### 4.8. Measuring Tumor-Infiltrating Lymphocytes (TILs) in Tumor Biopsies

Surgically obtained tumor samples were collected and fixed in 10% phosphate-buffered formaldehyde (formalin) for 24–48 h, dehydrated, embedded in paraffin, and underwent pathological examination for diagnosis. Hematoxylin and eosin (H&E) stained sections were used for morphological evaluation purposes and unstained sections for IHC studies. IHC staining was carried out according to standard methods. Briefly, 5-µm FFPE sections were deparaffinized and hydrated with serial passage through changes of xylene and graded ethanol. Sections were then subjected to heat-induced epitope retrieval with 0.01 M citrate buffer (pH 6.0). IHC was used to differentiate immune cell subsets, such as CD3+ (total T lymphocytes), CD4+ (helper T cells), CD8+ (cytotoxic T cells), FoxP3 (nuclear protein expresses by T regulatory cells), CD20+ (B cells), and CD68+ (macrophages). The following antibodies were used as follows: anti-CD3 pre-dilute (clone 2GV6; Ventana/Roche, Clifton, New Jersey, USA), incubation 32 min at 37 °C; anti-CD4 pre-dilute (clone sp35, Ventana/Roche, USA), incubation 16 min at 37 °C; anti-CD8 pre-dilute (clone sp57, Ventana/Roche, USA), incubation 16 min at 37 °C; anti-CD20 concentrate (Dako/Agilent, Santa Clara, CA, USA) (dilution 1:1280 in Ventana antibody dilution buffer), incubation 32 min at 37 °C; anti-CD68 (clone PG-M1, Dako/Agilent, Santa Clara, CA, USA) at 1:100 dilution; and anti-FoxP3 (D2W8ETM, Cell Signaling, Danvers, MA, USA) at 1:30 dilution in EDTA antigen retrieval. For each analysis, evaluation was conducted in the tumor and immediate peri-tumoral areas with quantitation as a percentage of positive cells relative to total nucleated cells. Scoring was conducted independently by three qualified pathologists (YG, RN, and KQC).

### 4.9. PD-1 and PD-L1 Expression in Tumor Biopsies

The anti-PD-L1, clone SP142 (Ventana/Roche, Clifton, New Jersey, USA) and, anti-PD-1 clone NAT105 (Cell Marque, Rocklin, CA, USA) were used for these IHCs. Stained slides were counterstained with hematoxylin and cover-slipped for review. Both PD-L1 and PD-1 were evaluated as the percentage area of positive staining within tumor-infiltrating lymphocytes (TILs). Cases with PD-L1 < 1% were considered negative and ≥1% were considered positive. Scoring was conducted independently by three board-certified pathologists (CS, RN, and YG).

### 4.10. Statistical Analysis

Comparisons between immune cell parameters were performed using Pearson and Wilcoxon rank-sum tests. P and R values for significance of the correlation between immune parameters were determined using a Spearman test. The two-sided log-rank test was used to compare overall survival (OS) between the TN and non-TN IBC groups, and patients with PDL-1 and PD-1 positive and negative tumors; Kaplan–Meier curves were plotted to visualize differences in survival profiles between these groups. Computations were performed in the R statistical language [51].

### 4.11. Data Availability

The datasets generated during and/or analyzed during the current study are available from the corresponding author on reasonable request.

## Figures and Tables

**Table 1:** Demographics and germline variants in IBC patients. IBC subtype, age at onset of the disease, race, overall survival (OS), and germline variants are indicated. Germline pathogenic (red), likely pathogenic (blue), and variants of uncertain significance (black). Normal tissue or whole blood samples were available for 20 patients (Pt-1 to Pt-15, Pt-19, Pt-20, Pt-21, Pt-24, and Pt-28) to confirm germline variants. † Putative germline variants. (1) Patient alive; (2) last record available. * Stop codon. Abbreviation: W, white; AA, African American; H, Hispanic; AJ, Ashkenazi Jewish heritage; TN, triple negative.

Patient ID	IBC Subtype	Age	Race	OS (months)	Gene	Variant Description	Protein Variant	Function
TN IBC								
Pt-1	TN	47	W	27	PALB2	c.1317delG	F440fs*12	Loss
Pt-2	TN	55	W	38	MSH2	c.1724A>G	D575G	Unknown
Pt-3	TN	46	W	17	MUTYH	c.1145G>A	G382D	Loss
Pt-4	TN	43	W (AJ)	42	BRCA1	c.68_69delAG	E23fs*17	Loss
Pt-5	TN	61	W	31	ESR1	c.805C>T	R269C	Loss
Pt-6	TN	44	W	19	BRCA2	c.7976G>A	R2659K	Loss
Pt-7	TN	48	W	27	KMT2C	c.10432C>G	Q3478E	Loss
Pt-8	TN	32	W	49 (2)	BARD1	c.2300_2301delTG	V767fs*4	Loss
Pt-9	TN	43	W	51	BRCA2	c.3975_3978dupTGCT	A1327fs*4	Loss
Pt-10	TN	45	AA	15	None			
Pt-11	TN	57	W	31	RAD51C	c.428A>G	Q143R	Loss
					AR	c.2395C>G	Q799E	Loss
Pt-12	TN (Her2+ at month 26)	44	W	34	None			
Pt-13	TN	42	W	24	FANCC	c.823T>C	F275L	Loss
Pt-14	TN	50	AA	34	None			
Pt-15	TN	43	W	17	None			
Pt-16	TN	43	AA	44	None			
Pt-17	TN	48	AA	50	None			
Pt-18	TN	61	W	32	None			
Non-TN IBC								
Pt-19	ER+ Her2−	34	W	76	ErbB2	c.2689C>T	R897W	Loss
Pt-20	ER+ Her2− (TN at month 8)	66	W	10	None			
Pt-21	ER+ Her2−	39	W	103 (1)	None			
Pt-22	ER+ Her2−	44	W	50	None			
Pt-23	ER+ Her2−	53	AA	6	BRCA1	c.5123C>T	A1708V	Loss
Pt-24	ER+ Her2−	50	W	41 (1)	ATM	c.6095G>A	R2032K	Loss
					APC	c.3386T>C	L1129S	Loss
Pt-25	ER+ Her2−	49	W	40	CHEK2	c.573+1G>A Splicing		Loss
Pt-26	ER+ Her2−	57	W	57 (1)	None			
Pt-27	ER+ Her2−	45	W	39	None			
Pt-28	ER+ Her2+ (ER+ Her2− at month 9)	56	W	21	BRCA2	c.9976A>T	K3326*	Loss
					MSH2	c.965G>A	G322D	Loss
Pt-29	ER+ Her2+ (ER− Her2+ at month 34)	32	W	44	^†^ ATM	c.4324T>C	Y1442H	Loss
					^†^ CASP8	c.152-1_155delGATTA		Loss
Pt-30	ER+ Her2+ (ER+ Her2− at month 49)	37	H	56	None			
Pt-31	ER− Her2+	69	Asian	37	^†^ IRAK4	c.529A>G	T177A	Loss
Pt-32	ER− Her2+	37	Asian	59	^†^ NCOR1	c.6641G>A	R2214H	Loss

**Table 2:** Immune cell infiltrates, PD-1 and PD-L1 expression in tumor tissue biopsies of IBC patients. The tumor tissue samples were quantified for total T lymphocytes (CD3+), helper T cells (CD4+), cytotoxic T cells (CD8+), B cells (CD20+), macrophages (CD68+), and T regulatory cells (FoxP3+). For each analysis, evaluation was conducted in the tumor and immediately peri-tumoral areas with quantitation as a percentage of positive cells relative to total nucleated cells. Both PD-L1 and PD-1 were evaluated as the percentage area of positive staining within tumor-infiltrating lymphocytes (TILs). PD-L1 < 1% was considered negative and >1% was considered positive; same for PD-1. PD-L1 and PD-1 positive values are bold. Overall survival (OS) is indicated for each patient. In OS: * Last record available, patient alive.

Patient ID	IBC Subtype	Tumor Tissue Sample	Total Lymphocytes	CD3	CD4+	CD8+	CD20	CD68	FOXP3+	PD-L1	PD-1	OS
			%	%	%	%	%	%	%	(% ITC positive)	(% ITC positive)	months
Pt-1	TN	punch biopsy	10	5	5	1	0	5	˂1	˂1	˂1	27
Pt-2	TN	punch biopsy	20	10	20	5	0	30	5	˂1	˂1	38
Pt-3	TN	chest wall, punch biopsy	10	10	20	15	5	25	10	**10**	**1**	17
Pt-5	TN	skin, chest wall	50	30	25	15	5	10	10	**1**	**5**	31
Pt-6	TN	skin, punch biopsy	5	5	10	5	0	10	1	˂1	˂1	19
Pt-7	TN	skin, punch biopsy	5	5	3	5	0	5	3	˂1	˂1	27
Pt-9	TN	skin, punch biopsy	40	20	15	10	3	25	15	˂1	˂1	51
Pt-11	TN	skin, punch biopsy	5	2	5	1	0	10	5	˂1	˂1	31
Pt-12	TN	skin, punch biopsy	10	10	10	5	0	30	5	˂1	˂1	34
Pt-14	TN	breast tissue	10	10	15	15	2	10	15	**10**	**10**	34
Pt-15	TN	skin, punch biopsy	20	15	20	5	˂1	15	3	**1**	**1**	17
Pt-17	TN	skin, punch biopsy	15	15	10	10	0	20	5	˂1	˂1	50
Pt-18	TN	skin, punch biopsy	10	5	10	10	0	5	3	˂1	˂1	32
Pt-19	ER+ Her2−	skin, biopsy	5	1	10	1	0	2	˂1	˂1	˂1	76
Pt-20	ER+ Her2− (TN at month 8)	skin, punch biopsy	10	10	15	5	˂1	15	2	˂1	˂ 1	10
Pt-24	ER+ Her2−	breast core biopsy	5	5	10	5	0	15	1	˂1	˂1	41 *
Pt-28	ER+ Her2+ (Her2− at month 9)	skin, punch biopsy	10	10	15	10	0	10	5	˂1	˂1	21
Pt-30	ER+ Her2+ (Her2− at month 49)	skin, punch biopsy	5	5	15	5	0	5	1	˂1	˂1	56
Pt-31	ER− Her2+	skin, punch biopsy	20	10	10	10	5	25	5	**1**	**1**	37
Pt-32	ER− Her2+	skin, punch biopsy	50	20	50	50	5	5	5	**1**	**1**	59
Pt-33	ER− Her2+	skin, punch biopsy	30	15	20	10	10	20	10	**1**	**5**	16
Pt-34	ER− Her2+	liver	30	20	30	10	1	20	5	˂1	˂1	127 *
Pt-35	TN/history of melanoma	skin, punch biopsy	50	30	50	10	0	15	5	˂1	˂1	47
Pt-36	ER− Her2+	skin, punch biopsy	10	20	15	10	0	20	5	˂1	˂1	76

## Supplementary Materials

The following are available online at https://www.mdpi.com/article/10.3390/ijms22168924/s1.

## Abbreviations

AF	allele fraction
AR	androgen receptor
cfDNA	cell-free DNA
ER	estrogen receptor
FFPE	formalin fixed paraffin embedded
Her2 (or ErbB2)	human epidermal growth factor receptor-2
HRR	homologous recombination repair
IBC	inflammatory breast cancer
IHC	immunohistochemistry
ICI	immune check point inhibitors
Indels	small insertions and deletions
mAb	monoclonal antibody
NGS	next generation sequencing
OCT	optimal cutting temperature compound
OS	overall survival
PARP	poly (ADP-ribose) polymerase
PD-1	programmed cell death protein 1
PD-L1	programmed cell death-ligand 1
PR	progesterone receptor
QCI	Qiagen Clinical Insight Interpret
RBC	red blood cells
SNVs	single nucleotide variants
TILs	tumor infiltrating lymphocytes
TN	triple negative
UMIs	unique molecular indices
VCF	variant call format

## References

[1] Cristofanilli M., Valero V., Buzdar A.U., Kau S.W., Broglio K.R., Gonzalez-Angulo A.M., Sneige N., Islam R., Ueno N.T., Buchholz T.A. (2007). Inflammatory breast cancer (IBC) and patterns of recurrence: Understanding the biology of a unique disease. Cancer.

[2] Lim B., Woodward W.A., Wang X., Reuben J.M., Ueno N.T. (2018). Inflammatory breast cancer biology: The tumour microenvironment is key. Nat. Rev. Cancer.

[3] Menta A., Fouad T.M., Lucci A., Le-Petross H., Stauder M.C., Woodward W.A., Ueno N.T., Lim B. (2018). Inflammatory Breast Cancer: What to Know About This Unique, Aggressive Breast Cancer. Surg. Clin. N. Am..

[4] Robertson F.M., Bondy M., Yang W., Yamauchi H., Wiggins S., Kamrudin S., Krishnamurthy S., Le-Petross H., Bidaut L., Player A.N. (2010). Inflammatory breast cancer: The disease, the biology, the treatment. CA Cancer J. Clin..

[5] Rowan K. (2009). Inflammatory breast cancer: New hopes and many hurdles. J. Natl. Cancer Inst..

[6] Levine P.H., Steinhorn S.C., Ries L.G., Aron J.L. (1985). Inflammatory breast cancer: The experience of the surveillance, epidemiology, and end results (SEER) program. J. Natl. Cancer Inst..

[7] Hance K.W., Anderson W.F., Devesa S.S., Young H.A., Levine P.H. (2005). Trends in inflammatory breast carcinoma incidence and survival: The surveillance, epidemiology, and end results program at the National Cancer Institute. J. Natl. Cancer Inst..

[8] Anderson W.F., Schairer C., Chen B.E., Hance K.W., Levine P.H. (2005). Epidemiology of inflammatory breast cancer (IBC). Breast Dis..

[9] Wingo P.A., Jamison P.M., Young J.L., Gargiullo P. (2004). Population-based statistics for women diagnosed with inflammatory breast cancer (United States). Cancer Causes Control.

[10] Bertucci F., Finetti P., Birnbaum D., Viens P. (2010). Gene expression profiling of inflammatory breast cancer. Cancer.

[11] Cakar B., Surmeli Z., Oner P.G., Yelim E.S., Karabulut B., Uslu R. (2018). The Impact of Subtype Distribution in Inflammatory Breast Cancer Outcome. Eur. J. Breast Health.

[12] Bertucci F., Finetti P., Vermeulen P., Van Dam P., Dirix L., Birnbaum D., Viens P., Van Laere S. (2014). Genomic profiling of inflammatory breast cancer: A review. Breast.

[13] Bertucci F., Rypens C., Finetti P., Guille A., Adelaide J., Monneur A., Carbuccia N., Garnier S., Dirix P., Goncalves A. (2020). NOTCH and DNA repair pathways are more frequently targeted by genomic alterations in inflammatory than in non-inflammatory breast cancers. Mol. Oncol..

[14] Bertucci F., Ueno N.T., Finetti P., Vermeulen P., Lucci A., Robertson F.M., Marsan M., Iwamoto T., Krishnamurthy S., Masuda H. (2014). Gene expression profiles of inflammatory breast cancer: Correlation with response to neoadjuvant chemotherapy and metastasis-free survival. Ann. Oncol..

[15] Masuda H., Baggerly K.A., Wang Y., Iwamoto T., Brewer T., Pusztai L., Kai K., Kogawa T., Finetti P., Birnbaum D. (2013). Comparison of molecular subtype distribution in triple-negative inflammatory and non-inflammatory breast cancers. Breast Cancer Res..

[16] Faldoni F.L.C., Villacis R.A.R., Canto L.M., Fonseca-Alves C.E., Cury S.S., Larsen S.J., Aagaard M.M., Souza C.P., Scapulatempo-Neto C., Osorio C. (2020). Inflammatory Breast Cancer: Clinical Implications of Genomic Alterations and Mutational Profiling. Cancers.

[17] Moslehi R., Freedman E., Zeinomar N., Veneroso C., Levine P.H. (2016). Importance of hereditary and selected environmental risk factors in the etiology of inflammatory breast cancer: A case-comparison study. BMC Cancer.

[18] Dawood S., Merajver S.D., Viens P., Vermeulen P.B., Swain S.M., Buchholz T.A., Dirix L.Y., Levine P.H., Lucci A., Krishnamurthy S. (2011). International expert panel on inflammatory breast cancer: Consensus statement for standardized diagnosis and treatment. Ann. Oncol..

[19] Fouad T.M., Barrera A.M.G., Reuben J.M., Lucci A., Woodward W.A., Stauder M.C., Lim B., DeSnyder S.M., Arun B., Gildy B. (2017). Inflammatory breast cancer: A proposed conceptual shift in the UICC-AJCC TNM staging system. Lancet Oncol..

[20] Fouad T.M., Kogawa T., Liu D.D., Shen Y., Masuda H., El-Zein R., Woodward W.A., Chavez-MacGregor M., Alvarez R.H., Arun B. (2015). Overall survival differences between patients with inflammatory and noninflammatory breast cancer presenting with distant metastasis at diagnosis. Breast Cancer Res. Treat..

[21] Ueno N.T., Espinosa Fernandez J.R., Cristofanilli M., Overmoyer B., Rea D., Berdichevski F., El-Shinawi M., Bellon J., Le-Petross H.T., Lucci A. (2018). International Consensus on the Clinical Management of Inflammatory Breast Cancer from the Morgan Welch Inflammatory Breast Cancer Research Program 10th Anniversary Conference. J. Cancer.

[22] Arias-Pulido H., Cimino-Mathews A., Chaher N., Qualls C., Joste N., Colpaert C., Marotti J.D., Foisey M., Prossnitz E.R., Emens L.A. (2018). The combined presence of CD20+ B cells and PD-L1+ tumor-infiltrating lymphocytes in inflammatory breast cancer is prognostic of improved patient outcome. Breast Cancer Res. Treat..

[23] Bertucci F., Finetti P., Colpaert C., Mamessier E., Parizel M., Dirix L., Viens P., Birnbaum D., van Laere S. (2015). PDL1 expression in inflammatory breast cancer is frequent and predicts for the pathological response to chemotherapy. Oncotarget.

[24] Hamm C.A., Moran D., Rao K., Trusk P.B., Pry K., Sausen M., Jones S., Velculescu V.E., Cristofanilli M., Bacus S. (2016). Genomic and Immunological Tumor Profiling Identifies Targetable Pathways and Extensive CD8^+^/PDL1^+^ Immune Infiltration in Inflammatory Breast Cancer Tumors. Mol. Cancer Ther..

[25] Van Berckelaer C., Rypens C., van Dam P., Pouillon L., Parizel M., Schats K.A., Kockx M., Tjalma W.A.A., Vermeulen P., van Laere S. (2019). Infiltrating stromal immune cells in inflammatory breast cancer are associated with an improved outcome and increased PD-L1 expression. Breast Cancer Res..

[26] Fernandez S.V., MacFarlane A.W.T., Jillab M., Arisi M.F., Yearley J., Annamalai L., Gong Y., Cai K.Q., Alpaugh R.K., Cristofanilli M. (2020). Immune phenotype of patients with stage IV metastatic inflammatory breast cancer. Breast Cancer Res..

[27] Winn J.S., Hasse Z., Slifker M., Pei J., Arisi-Fernandez S.M., Talarchek J.N., Obeid E., Baldwin D.A., Gong Y., Ross E. (2020). Genetic Variants Detected Using Cell-Free DNA from Blood and Tumor Samples in Patients with Inflammatory Breast Cancer. Int. J. Mol. Sci..

[28] Breast Cancer Association Consortium (2021). Breast Cancer Risk Genes—Association Analysis in More than 113,000 Women. N. Engl. J. Med..

[29] Win A.K., Dowty J.G., Cleary S.P., Kim H., Buchanan D.D., Young J.P., Clendenning M., Rosty C., MacInnis R.J., Giles G.G. (2014). Risk of colorectal cancer for carriers of mutations in MUTYH, with and without a family history of cancer. Gastroenterology.

[30] Wasielewski M., Out A.A., Vermeulen J., Nielsen M., van den Ouweland A., Tops C.M., Wijnen J.T., Vasen H.F., Weiss M.M., Klijn J.G. (2010). Increased MUTYH mutation frequency among Dutch families with breast cancer and colorectal cancer. Breast Cancer Res. Treat..

[31] Rennert G., Lejbkowicz F., Cohen I., Pinchev M., Rennert H.S., Barnett-Griness O. (2012). MutYH mutation carriers have increased breast cancer risk. Cancer.

[32] Litton J.K., Rugo H.S., Ettl J., Hurvitz S.A., Goncalves A., Lee K.H., Fehrenbacher L., Yerushalmi R., Mina L.A., Martin M. (2018). Talazoparib in Patients with Advanced Breast Cancer and a Germline BRCA Mutation. N. Engl. J. Med..

[33] Robson M., Im S.A., Senkus E., Xu B., Domchek S.M., Masuda N., Delaloge S., Li W., Tung N., Armstrong A. (2017). Olaparib for Metastatic Breast Cancer in Patients with a Germline BRCA Mutation. N. Engl. J. Med..

[34] Rana H.Q., Sacca R., Drogan C., Gutierrez S., Schlosnagle E., Regan M.M., Speare V., LaDuca H., Dolinsky J., Garber J.E. (2019). Prevalence of germline variants in inflammatory breast cancer. Cancer.

[35] Daly M.B., Pilarski R., Berry M., Buys S.S., Farmer M., Friedman S., Garber J.E., Kauff N.D., Khan S., Klein C. (2017). NCCN Guidelines Insights: Genetic/Familial High-Risk Assessment: Breast and Ovarian, Version 2.2017. J. Natl. Compr. Cancer Netw..

[36] Pujade-Lauraine E., Ledermann J.A., Selle F., Gebski V., Penson R.T., Oza A.M., Korach J., Huzarski T., Poveda A., Pignata S. (2017). Olaparib tablets as maintenance therapy in patients with platinum-sensitive, relapsed ovarian cancer and a BRCA1/2 mutation (SOLO2/ENGOT-Ov21): A double-blind, randomised, placebo-controlled, phase 3 trial. Lancet Oncol..

[37] McCabe N., Turner N.C., Lord C.J., Kluzek K., Bialkowska A., Swift S., Giavara S., O’Connor M.J., Tutt A.N., Zdzienicka M.Z. (2006). Deficiency in the repair of DNA damage by homologous recombination and sensitivity to poly(ADP-ribose) polymerase inhibition. Cancer Res..

[38] Buisson R., Dion-Cote A.M., Coulombe Y., Launay H., Cai H., Stasiak A.Z., Stasiak A., Xia B., Masson J.Y. (2010). Cooperation of breast cancer proteins PALB2 and piccolo BRCA2 in stimulating homologous recombination. Nat. Struct. Mol. Biol..

[39] Kondrashova O., Nguyen M., Shield-Artin K., Tinker A.V., Teng N.N.H., Harrell M.I., Kuiper M.J., Ho G.Y., Barker H., Jasin M. (2017). Secondary Somatic Mutations Restoring RAD51C and RAD51D Associated with Acquired Resistance to the PARP Inhibitor Rucaparib in High-Grade Ovarian Carcinoma. Cancer Discov..

[40] Calderaro J., Petitprez F., Becht E., Laurent A., Hirsch T.Z., Rousseau B., Luciani A., Amaddeo G., Derman J., Charpy C. (2019). Intra-tumoral tertiary lymphoid structures are associated with a low risk of early recurrence of hepatocellular carcinoma. J. Hepatol..

[41] Fridman W.H., Zitvogel L., Sautes-Fridman C., Kroemer G. (2017). The immune contexture in cancer prognosis and treatment. Nat. Rev. Clin. Oncol..

[42] Borst J., Ahrends T., Babala N., Melief C.J.M., Kastenmuller W. (2018). CD4^+^ T cell help in cancer immunology and immunotherapy. Nat. Rev. Immunol..

[43] Francisco L.M., Sage P.T., Sharpe A.H. (2010). The PD-1 pathway in tolerance and autoimmunity. Immunol. Rev..

[44] Patel S.P., Kurzrock R. (2015). PD-L1 Expression as a Predictive Biomarker in Cancer Immunotherapy. Mol. Cancer Ther..

[45] Spranger S., Spaapen R.M., Zha Y., Williams J., Meng Y., Ha T.T., Gajewski T.F. (2013). Up-regulation of PD-L1, IDO, and T_regs_ in the melanoma tumor microenvironment is driven by CD8^+^ T cells. Sci. Transl. Med..

[46] Jiao S., Xia W., Yamaguchi H., Wei Y., Chen M.K., Hsu J.M., Hsu J.L., Yu W.H., Du Y., Lee H.H. (2017). PARP Inhibitor Upregulates PD-L1 Expression and Enhances Cancer-Associated Immunosuppression. Clin. Cancer Res..

[47] Veneris J.T., Matulonis U.A., Liu J.F., Konstantinopoulos P.A. (2020). Choosing wisely: Selecting PARP inhibitor combinations to promote anti-tumor immune responses beyond BRCA mutations. Gynecol. Oncol..

[48] Domchek S.M., Postel-Vinay S., Im S.A., Park Y.H., Delord J.P., Italiano A., Alexandre J., You B., Bastian S., Krebs M.G. (2020). Olaparib and durvalumab in patients with germline BRCA-mutated metastatic breast cancer (MEDIOLA): An open-label, multicentre, phase 1/2, basket study. Lancet Oncol..

[49] Li M.M., Datto M., Duncavage E.J., Kulkarni S., Lindeman N.I., Roy S., Tsimberidou A.M., Vnencak-Jones C.L., Wolff D.J., Younes A. (2017). Standards and Guidelines for the Interpretation and Reporting of Sequence Variants in Cancer: A Joint Consensus Recommendation of the Association for Molecular Pathology, American Society of Clinical Oncology, and College of American Pathologists. J. Mol. Diagn..

[50] Richards S., Aziz N., Bale S., Bick D., Das S., Gastier-Foster J., Grody W.W., Hegde M., Lyon E., Spector E. (2015). Standards and guidelines for the interpretation of sequence variants: A joint consensus recommendation of the American College of Medical Genetics and Genomics and the Association for Molecular Pathology. Genet. Med..

[51] R Foundation for Statistical Computing (2018). A Language and Environment for Statistical Computing.

