# Supplementary material for: Genetic Variants and Tumor Immune Microenvironment: Clues for Targeted Therapies in Inflammatory Breast Cancer (IBC)
Source: Int J Mol Sci. 2021 Aug 19;22(16):8924. doi: 10.3390/ijms22168924 (PMC8396191; doi:10.3390/ijms22168924)
Supplement: Supplementary file 1 [file ijms-22-08924-s001.zip › Table S1.pdf]

| Patient ID | Tissue biopsy | Liquid biopsy |
|---|---|---|
| Pt-1 | Skin biopsy, right scapula- Month 12 (Stage IV) | Plasma (cfDNA2) - Month 22 (Stage IV) |
| | **PALB2 c.1317delG; p. F440fs*12 (43%) Loss/T2C | **PALB2 c.1317delG; p. F440fs*12 (51%) Loss/T2C<br>RB1 splice 607+1 G>C (11%) Loss/T2C |
| Pt-2 | Breast tissue biopsy - Month 33 (Stage IV) | Serum- Month 33 (Stage IV) |
| | **MSH2 c.1724A>G; p. D575G (51%) Normal<br>TP53 c.721_722insA; p. S241fs*23 (16%) Loss/T2C | **MSH2 c.1724A>G; p. D575G (44%) Unknown<br>TP53 c.721_722insA; p. S241fs*23 (5.59%) Loss/T2C |
| Pt-3 | Cells from malignant pleural effusion -Month 12 (Stage IV) | Serum - Month 12 (Stage IV) |
| | **MUTYH c.1145G>A; p. G382D (44%) Loss/T3<br>TP53 c.375+1G>T (21%) Loss/T2C | **MUTYH c.1145G>A; p. G382D (43%) Loss/T3<br>TP53 c.375+1G>T (56%) Loss/T2C<br>PMS2 c.89A>C; p.Q30P (2.97%) Loss/T2C |
| Pt-4 | Skin breast biopsy- Month 17 (Stage IV) | Serum - Month 17 (Stage IV) |
| | **BRCA1 c.68_69delAG; p. E23fs*17 (50%) Loss/T1A | **BRCA1 c.68_69delAG; p. E23fs*17 (48%) Loss/T1A<br>MSH6 c.3261delC; p. F1088fs*2 (2.92%) Loss/T2C |
| Pt-5 | Skin upper arm biopsy- Month 13 (Stage IV) | Serum- Month 13 (Stage IV) |
| | **ESR1 c.805C>T; p. R269C (48%) Loss/T3 | **ESR1 c.805C>T; p. R269C (50%) Loss/T3 |
| Pt-6 | Breast tissue- Month 3 (Stage IV) | Serum - Month 3 (Stage IV) |
| | ***BRCA2 c.7976G>A; p. R2659K (50%) Loss/T1A<br>MEN1 c.1471G>T; p. E491* (71%) Loss/T3 | ***BRCA2 c.7976G>A; p. R2659K (50%) Loss/T1A<br>MRE11 c.1532delA; p. N511fs*13 (2.64%) Loss/T2C |
| Pt-7 | Cells malignant pleural effusion-Month 25 (Stage IV) | Plasma- Month 21 (Stage IV) |
| | **KMT2C c.10432C>G; p. Q3478E (43%) Loss/T3<br>TP53 c.817C>T; p. R273C (58%) T2C | **KMT2C c.10432C>G; p. Q3478E (50%) Loss/T3<br>TP53 c.817C>T; p. R273C (6.77%) T2C<br>MSH6 c.3261delC; p. F1088fs*2 (2.63%) Loss/T2C |
| Pt-8 | Skin biopsy breast- Month 8 (Stage IV) | Plasma- Month 15 (Stage IV) |
| | **BARD1 c.2300_2301delTG; p. V767fs*4 (44%) Loss/T3<br>AR c.170T>A; p. L57Q (4.86%) Loss/T3 | **BARD1 c.2300_2301delTG; p. V767fs*4 (41%) Loss/T3 |
| Pt-9 | Skin biopsy- Month 28 (Stage IV) | Plasma- Month 30 (Stage IV) |
| | **BRCA2 c.3975_3978dupTGCT; p. A1327fs*4 (50%) Loss/T1A | **BRCA2 c.3975_3978dupTGCT; p. A1327fs*4 (59%) Loss/T1A<br>TP53 c.328delC; p. R110fs*13 (12%) Loss/T2C<br>RB1 c.2158_2164delAAATTCAinsTGTAACAT; p.K720fs*31 (9.4%) |

| | | |
|---|---|---|
| | | Loss/T3 |
| Pt-10 | Lymph node- Month 9 (Stage IV) | Plasma - Month 9 (Stage IV) |
| | TP53 c.329delG; p. R110fs*13 (74%) Loss/T2C | TP53 c.329delG; p. R110fs*13 (19%) Loss/T2C |
| Pt-11 | Skin from chest wall- Month 21 (Stage IV) | Plasma- Month 23 (Stage IV) |
| | **RAD51C c.428A>G; p. Q143R (79%) Loss/T2C<br>**AR c.2395C>G; p. Q799E (72%) Loss/T3<br>TP53 c.686_687delGT; p.C229fs*10 (48%) Loss/T2C<br>ERBB2 c.2329G>T; p. V777L (54%) Gain/T2C<br>ERBB2 c.929C>T; p. S310F (55%) Gain/T2C<br>PIK3CA c.331A>G; p. K111E (38%) Gain/T2C | **RAD51C c.428A>G; p. Q143R (56%) Loss/T2C<br>**AR c.2395C>G; p. Q799E (51 %) Loss/T3<br>TP53 c.686_687delGT; p. C229fs*10 (13%) Loss/T2C<br>ERBB2 c.2329G>T; p. V777L (6.71%) Gain/T2C<br>ERBB2 c.929C>T; p. S310F (9.59%) Gain/T2C<br>PIK3CA c.331A>G; p. K111E (9.05%) Gain/T2C |
| Pt-12 | Skin biopsy- Month 27 (Stage IV) | Serum - Month 27 (Stage IV) |
| | PTEN c.30_41delCAGAAACAAAAG; p. S10_K13del (69%) Loss/T2C<br>PMS1 c.2153T>G; p. L718* (80%) Loss/T2C<br>BAP1 c.680G>C; p. R227P (72%) Loss/T2C | PTEN c.30_41delCAGAAACAAAAG; p. S10_K13del (6.1%) Loss/T2C<br>PMS1 c.2153T>G; p. L718* (4.9%) Loss/T2C<br>BAP1 c.680G>C; p. R227P (2.84%) Loss/T2C<br>TP53 c.292_315delCCTTCCCAGAAAACCTACCAGGGCinsAG<br>p. P98fs*18 (5.29% of 1438) T2C |
| Pt-13 | NO tumor tissue available | Plasma- Month 9 (Stage IV) |
| | | **FANCC c.823T>C; p. F275L (55%) Loss/T3<br>TP53 c.499C>T; p. Q167* (5.06%) Loss/T2C<br>MSH6 c.3261delC; p. F1088fs*2 (2%) Loss/T2C |
| Pt-14 | Breast tissue- Month 7 (Stage III) | Plasma – Month 10 (Stage III) |
| | PTEN c.968dupA; p. N323fs*2 (12%) Loss/T2C<br>PIK3R1 c.1102delT; p. Y368fs*5 (12%) T2C<br>PIK3R1 c.1709_1735delTTATCCAGCTGAGAAAGACGAGAGACC<br>p. L570_D578del (6.89%) T2C | PTEN c.968dupA; p. N323fs*2 (3.95%) Loss/T2C<br>AR c.170T>A; p. L57Q (3.44%) Loss T3<br>KMT2C c.906T>A; p.C302* (4.19%) Loss/T3<br>PIK3R1 c.1362_1364dupTCA; p. T454_Q455insH (3.04%) Loss/T2C |
| Pt-15 | Skin breast biopsy- Month 1 (Stage III) | Plasma - Month 1 (Stage III) |
| | TP53 c.375G>C; p. T125T (3.37%) Loss/T3 | TP53 c.375G>C; p. T125T (30%) Loss/T3 |
| Pt-16 | Cells from malignant fluid abdomen - Month 44 (Stage IV) | Serum- Month 44 (Stage IV) |
| | RB1 c.2336T>A; p. L779* (89%) Loss/T3<br>TP53 c.294_310delTTCCCAGAAAACCTACC; p. S99fs*44 (83%) | RB1 c.2336T>A; p. L779* (24%) Loss/T3<br>TP53 c.294_310delTTCCCAGAAAACCTACC; p. S99fs*44 (23%) |

| | | |
|---|---|---|
| | Loss/T2C<br>PMS2 c.1239delA; p. D414fs*34 (2%) Loss/T2C | Loss/T2C<br>PMS2 c.1239delA; p. D414fs*34 (2.5%) Loss/T2C |
| Pt-17 | **Skin chest wall- Month 35 (Stage IV)** | **Serum- Month 35 (Stage IV)** |
| | TP53 c.690_702delCACCATCCACTAC; p. I232fs*11 (56%) Loss/T2C<br>BRCA1 c.5278-1G>T (33%) Loss/T1A | TP53 c.690_702delCACCATCCACTAC; p. I232fs*11 (21%) Loss/T2C<br>BRCA1 c.5278-1G>T (9.1%) Loss/T1A<br>PMS2 c.89A>C; p. Q30P (2%) T2C |
| Pt-18 | **Breast tumor tissue- Month 15 (Stage IV)** | **Plasma- Month 15 (Stage IV)** |
| | *RETc.2071G>A; p. G691S (99%) Gain/T3<br>TP53 c.1024C>T; p. R342* (41%) Loss/T2C<br>EGFR c.2281G>A; p. D761N (3.2%) Gain/T2C<br>ATM c.8287C>T; p. R2763* (2.1%) Loss/T2C (2.1%) | *RETc.2071G>A; p. G691S (100%) Gain/T3<br>TP53 c.1024C>T; p. R342* (3.21%) Loss/T2C |

**Table S1. Germline and somatic variants in triple negative IBC.** Clinically relevant germline and somatic variants are shown. Time of collection from disease onset and stage of the disease at the time of sample collection are indicated in each case. For each variant, its allele fraction (AF) is indicated in parenthesis. The variants were classified according their pathogenicity as pathogenic (in red), likely pathogenic (in blue), or variants of uncertain significance (in black). Variants were also classified according to its actionability: T1A indicates strong clinical significance; T2C indicates potential clinical significance and FDA-approved therapies for different tumor types or investigational therapies; T3 indicates biomarker is of uncertain clinical significance. Loss or gain of protein functions are indicated. **Germline variants confirmed by NGS of whole blood or normal tissue. ***Germline variant according to clinical test in patient clinical history file. For Pt-1, cfDNA samples from month 12 (cfDNA1) and month 22 (cfDNA2) were studied, and *PALB2* variant was detected in both, but the *RB1* mutation was only detected in cfDNA from month 22. Protein loss or gain of function is indicated for some variants.
