# Supplementary material for: Genetic Variants and Tumor Immune Microenvironment: Clues for Targeted Therapies in Inflammatory Breast Cancer (IBC)
Source: Int J Mol Sci. 2021 Aug 19;22(16):8924. doi: 10.3390/ijms22168924 (PMC8396191; doi:10.3390/ijms22168924)
Supplement: Supplementary file 1 [file ijms-22-08924-s001.zip › Table S2.pdf]

Patient ID	Tissue biopsy	Liquid biopsy
Pt-19	Skin biopsy- Month 54 (Stage IV)	Plasma - Month 54 (Stage IV)
	**ErbB2 c.2689C>T; p. R897W (56%) Loss/T3<br>PIK3CA c.3140A>G; p.H1047R (48%) Gain/T2C<br>ESR1 c.1613A>G; p. D538G (22%) Gain/T1B	**ErbB2 c.2689C>T; p. R897W (52%) Loss/T3
Pt-20	Skin chest biopsy - Month 8 (Stage IV)	Plasma- Month 8 (Stage IV)
	TP53 c.796G>A; p. G266R (48%) Loss/T2C<br>MRE11 c.1532delA; p. N511fs*13 (2.1%) Loss/T2C<br>RB1 c.131_132insTT; p. V45fs*21 (43%) Loss/T3	TP53 c.796G>A; p. G266R (48%) Loss/T2C<br>AR c.170T>A; p. L57Q (4.06%) Loss/T3<br>RB1 c.131_132insTT; p. V45fs*21 (50%) Loss/T3
Pt-21	Cells pleural fluid - Month 9 (Stage IV)	Plasma- Month 3 (Stage IV)
	Not pathogenic or likely path variants detected	MAP2K4 c.400C>T; p. R134W (26%) Gain/T3<br>PMS2 c.89A>C; p. Q30P (2%) Loss/T2C<br>MSH6 c.3261delC; p. F1088fs*2 (2%) Loss/T2C
Pt-22	Cells pleural fluid- Month 29 (Stage IV)	Serum- Month 29 (Stage IV)
	TP53 c.818G>A; p. R273H (30%) T2C<br>NCOR1 c.842+1G>A (12%) Loss/T3<br>PTEN c.955_958delACTT; p. T319* (9.83%) Loss/T2C<br>PTEN c.843_858delAGGACCAGAGGAAACC<br>p.G282fs*4 (8.1%) Loss/T2C	TP53 c.818G>A; p. R273H (2%) T2C
Pt-23	Cells pleural fluid- Month 6 (Stage IV)	Serum - Month 6 (Stage IV)
	BRCA1 c.5123C>T; p. A1708V (49%) Loss/T1A<br>TP53 c.637C>T; p. R213(12%) Loss/T2C	*BRCA1 c.5123C>T; p. A1708V (54%) Loss/T1A<br>TP53 c.637C>T; p. R213*(11%) Loss/T2C
Pt-24	Breast biopsy- Month 5 (Stage IIIC)	Plasma- Month 9 (Stage IIIC)
	**ATM c.6095G>A; p. R2032K (53%) Loss/T2C<br>**APC c.3386T>C; p. L1129S (48%) Loss/T3<br>PIK3CA c.3140A>G; p.H1047R (4.8%) Gain/T1A	**ATM c.6095G>A; p. R2032K (51%) T2C<br>**APC c.3386T>C; p. L1129S (51%) Loss/T3
Pt-25	Skin biopsy- Month 19 (Stage III)	Serum- Month 19 (Stage III)
	*CHEK2 c.573+1G>A Splicing (50%) Loss/T2C<br>TP53 c.286dupT; p. S96fs*53 (4.88%) Loss/T2C	*CHEK2 c.573+1G>A (46%) Splicing Loss/T2C

Pt-26	**Breast biopsy-Month 1 (Stage IIIB)**	**Serum (cfDNA2)- Month 1 (Stage IIIB)**
	**MSH6 c.3261delC; p. F1088fs*2 (2.28%) Loss/T2C**	**MSH6 c.3261delC; p. F1088fs*2 (3.09%) Loss/T2C**
Pt- 27		**Plasma- Month 1 (Stage IIIC)**
	No tissue available	**MSH6 c.3261delC; p. F1088fs*2 (2.27%) Loss/T2C**

Table S2. Germline and somatic variants in ER+ Her2- IBC. Clinically relevant germline and somatic variants are shown. Pathogenic (in red), likely-pathogenic (in blue) or variants of uncertain significance (in black). **Confirmed germline variants. *Putative germline variants. Variants were also classified according to their actionability: T1A/T1B indicates strong clinical significance; T2C indicates potential clinical significance and FDA-approved therapies for different tumor types or investigational therapies (off-label treatments); T3 indicates biomarker is of uncertain clinical significance. Stage of the disease at the time of sample collection is indicated in each case. For each variant, allele fraction (AF) is indicated in parenthesis.
