# Supplementary material for: Genetic Variants and Tumor Immune Microenvironment: Clues for Targeted Therapies in Inflammatory Breast Cancer (IBC)
Source: Int J Mol Sci. 2021 Aug 19;22(16):8924. doi: 10.3390/ijms22168924 (PMC8396191; doi:10.3390/ijms22168924)
Supplement: Supplementary file 1 [file ijms-22-08924-s001.zip › Table S3.pdf]

Patient ID	Tissue biopsy or cells from pleural effusion	Liquid biopsy
	Skin biopsy- Month 15 (Stage IV)	Serum- Month 15 (cfDNA2) (Stage IV)
Pt-28	**BRCA2 c.9976A>T; p. K3326* (65%) Loss/T3<br>**MSH2 c.965G>A; p. G322D (56%) Loss/T3<br>PTEN c.319G>T; p. D107Y (20%) Loss/T2C<br>RB1 c.1959dupA; p. V654fs*14 (6.26%) Loss/T3<br>TP53 c.375G>T; p. T125T (15%) Loss/T3	**BRCA2 c.9976A>T; p. K3326* (57%) Loss/T3<br>**MSH2 c.965G>A; p. G322D (56%) Loss/T3<br>PTEN c.319G>T; p. D107Y (8.3%) Loss/T2C<br>RB1 c.1959dupA; p. V654fs*14 (1.7%) Loss/T3<br>TP53 c.375G>T; p. T125T (6.2 %) Loss/T3
Pt-29	Cells pleural fluid- Month 33 (Stage IV)	Serum - Month 33 (Stage IV)
	*ATM c.4324T>C; p. Y1442H (53%) Loss/T3<br>*CASP8 c.152-1_155delGATTA (51%) Loss/T3<br>PALB2 c.2257C>T; p. R753* (4.17%) Loss/T2C<br>ERCC4 c.1243C>T; p. R415* (5%) Loss/T3<br>HERC1 c.6790C>T; p. R2264*(4.44%) Loss/T3	*ATM c.4324T>C; p. Y1442H (52%) Loss/T3<br>*CASP8 c.152-1_155delGATTA (49%) Loss/T3
Pt-30	Cells pleural effusion- Month 50 (Stage IV)	Serum - Month 50 (Stage IV)
	PMS2 c.1239delA; p. D414fs*34 (2.3%) Loss/T2C	MED12 c.6338_6339insCCA; p. Q2112_Q2113insH (3.3%) T3
Pt-31	Skin biopsy breast- Month 31 (Stage IV)	Plasma -Month 25 (Stage IV)
	*IRAK4 c.529A>G; p. T177A (61%) Loss/T3<br>TP53 c.602delT; p. L201fs*46 (57%) Loss/T2C	*IRAK4 c.529A>G; p. T177A (57%)<br>TP53 c.602delT; p. L201fs*46 (16%) Loss/T2C<br>TP53 c.638G>A; p. R213Q (4.8%) Loss/T2C
Pt-32		Plasma- Month 8 (Stage IIIB)
	No tissue available	*NCOR1 c.6641G>A; p. R2214H (48%) Loss/T3

Table S3. Germline and somatic variants in Her2+ IBC patients. Clinically relevant variants are shown. **Germline variants that were confirmed by NGS of normal tissue or whole blood. *Putative germline variants. In red, pathogenic variants; blue, likely pathogenic; in black, variants from uncertain significance. Variants were also classified according their actionability: T2C indicates potential clinical significance and FDA-approved therapies for different tumor types or investigational therapies (off- label treatments); T3 indicates biomarker is of uncertain clinical significance. Stage of the disease at the time of sample collection is indicated in each case.
