# Supplementary material for: Genetic Variants and Tumor Immune Microenvironment: Clues for Targeted Therapies in Inflammatory Breast Cancer (IBC)
Source: Int J Mol Sci. 2021 Aug 19;22(16):8924. doi: 10.3390/ijms22168924 (PMC8396191; doi:10.3390/ijms22168924)
Supplement: Supplementary file 1 [file ijms-22-08924-s001.zip › Table S4.pdf]

ACVR1B	CBFB	ERCC4	KMT2C	NCOR1	RAD51	XRCC2
AKT1	CCND1	ESR1	KRAS	NEK2	RAD51C	XRCC3
APC	CDH1	EXOC2	MAP2K4	NF1	RAD51D	ZBED4
AR	CDK4	EXT2	MAP3K1	PALB2	RB1	
ATM	CDK6	FAM175A	MDM2	PALLD	RET	
ATR	CDKN2A	FANCC	MED12	PBRM1	SEPT9	
AXIN2	CHEK2	FBXO32	MEN1	PCGF2	SMAD4	
BAP1	CSMD1	FGFR1	MLH1	PIK3CA	SMARCA4	
BARD1	CTNNB1	FGFR2	MRE11A	PIK3R1	STK11	
BLM	DIRAS3	GATA3	MSH2	PMS1	SYNE1	
BMPR1A	EGFR	GEN1	MSH6	PMS2	TGFB1	
BRCA1	EP300	HERC1	MUC16	PPM1L	TP53	
BRCA2	EPCAM	HOXB13	MUTYH	PTEN	TRAF5	
BRIP1	ERBB2	IRAK4	MYC	PTGFR	VHL	
CASP8	ERBB3	ITCH	NBN	RAD50	WEE1	

Table S4. Breast cancer panel DHS-001Z (Qiagen). The breast cancer panel used in this study covers 93 genes and is able to detect single nucleotide variants (SNV) and small insertions and deletions (indels). Copy number changes were not studied.
