# Supplementary material for: Genetic Variants and Tumor Immune Microenvironment: Clues for Targeted Therapies in Inflammatory Breast Cancer (IBC)
Source: Int J Mol Sci. 2021 Aug 19;22(16):8924. doi: 10.3390/ijms22168924 (PMC8396191; doi:10.3390/ijms22168924)
Supplement: Supplementary file 1 [file ijms-22-08924-s001.zip › Table S6.pdf]

	cfDNA			DNA from tumor (tissue or malignant cells from pleural effusion or ascites)			DNA from normal tissue or whole blood (to study germline variants)		
Patient ID	Quantity to prepare libraries	Total number of reads	Mean read depth	Quantity of DNA to prepare libraries	Total number of reads	Mean read depth	Quantity of DNA to prepare libraries	Total number of reads	Mean read depth
Pt-001	13 ng (plasma) Month 12	4,438,780	1,450.76	17 ng (skin biopsy; FFPE) Month 12	3,761,410	1,270.41	129 ng (normal tissue; FFPE)	10,337,397	3,459.28
Pt-002	19 ng (serum) Month 33	3,942,080	1,279.20	80 ng (breast tissue; OCT) Month 33	3,754,267	1,270.41	151 ng (whole blood)	9,066,325	2,606.33
Pt-003	19 ng (serum) Month 12	3,222,292	1,073.01	80 ng (pleural effusion; OCT) Month 12	3,254,060	1,122.18	232 ng (normal tissue; FFPE)	11,348,728	3,878.66
Pt-004	30 ng (serum) Month 17	4,987,114	718.06	310 ng (skin breast biopsy; FFPE) Month 17	7,714,165	2,673.43	298 ng (normal tissue; FFPE)	9,385,812	2,665.22
Pt-005	10 ng (serum) Month 13	4,176,953	355.97	417 ng (skin upper arm; OCT) Month 13	8,671,157	2,918.10	150 ng (whole blood)	9,346,793	3,207.81
Pt-006	45 ng (serum) Month 3	4,967,670	1,764.53	300 ng (breast tissue; OCT) Month 3	8,297,537	2,816.02	Genetic clinical testing show BRCA2 germline variant		
Pt-007	80 ng (plasma) Month 21	3,391,998	1,115.87	80 ng (pleural effusion; OCT) Month 26	3,872,538	1,281.61	181 ng (whole blood)	8,026,668	3,035.35
Pt-008	30 ng (plasma) Month 15	6,088,580	689.97	301 ng (skin breast biopsy; OCT) Month 8	6,700,776	2,335.01	155 ng (whole blood)	7,656,821	1,425.38
Pt-009	17 ng (plasma) Month 30	4,282,461	1,544.24	425 ng (skin biopsy; FFPE) Month 28	15,200,246	5,285.77	177 ng (whole blood)	8,087,635	2,644.36

Pt-010	31 ng (plasma) Month 9	4,673,532	1,486.47	279 ng (lymph node; FFPE) Month 9	11,033,228	3,538.95	171 ng (whole blood)	8,686,525	2,990.95
Pt-011	12 ng (plasma) Month 23	3,710,359	1,344.14	182 ng (skin chest wall; FFPE) Month 21	9,968,580	3,484.52	269 ng (whole blood)	7,685,845	2,763.74
Pt-012	21 ng (serum) Month 27	5,889,286	1,969.86	254 ng (skin biopsy; FFPE) Month 27	9,539,497	3,271.85	260 ng (normal tissue; FFPE)	10,117,393	3,193.36
Pt-013	13 ng (plasma) Month 9	3,312,305	1,155.96	Not tumor tissue available	N/A	N/A	151 ng (whole blood)	8,511,404	2,713.51
Pt-014	111 ng (plasma) Month 10	4,621,711	1,478.06	259 ng (breast tissue; FFPE) Month 7	18,327,056	6,496.75	296 ng (whole blood)	7,756,132	2,743.80
Pt-015	17 ng (plasma) Month 1	3,878,882	1,303.97	331 ng (skin breast biopsy; OCT) Month 1	7,362,160	2,626.14	223 ng (whole blood)	8,429,943	3,069.48
Pt-016	101 ng (serum) Month 44	5,313,685	1,776.08	300 ng (ascites; OCT) Month 44	4,073,252	1,220.99	Not available		
Pt-017	23 ng (serum) Month 35	4,993,580	1,623.99	300 ng (skin chest wall; OCT) Month 35	4,460,607	1,435.68	Not available		
Pt-018	16 ng (plasma) Month 15	3,280,303	1,093.23	259 ng (breast tumor; FFPE) Month 15	8,187,575	1,661.08	Not available		
Pt-019	10 ng (plasma) Month 54	5,628,552	1,801.65	80 ng (skin biopsy; OCT) Month 54	8,752,734	2,328.37	155 ng (whole blood)	7,404,018	2,854.16
Pt-020	17 ng (plasma) Month 8	5,331,490	610.27	300 ng (skin chest biopsy; OCT) Month 8	7,193,646	2,536.12	149 ng (whole blood)	8,020,579	2,965.83
Pt-021	39 ng	3,868,785	1,240.69	80 ng	3,900,340	1,347.22	227.2 ng	7,806,273	2,937.94

	(plasma)<br>Month 3			(pleural fluid; FFPE)<br>Month 9			(whole blood)		
Pt-022	10 ng<br>(serum)<br>Month 29	5,081,025	532.79	300 ng<br>(pleural fluid; OCT)<br>Month 29	5,580,422	1,854.43	Not available		
Pt-023	335 ng<br>(serum)<br>Month 6	4,834,788	1,519.55	138 ng<br>(pleural fluid; OCT)<br>Month 6	9,133,646	3,171.36	Not available		
Pt-024	73 ng<br>(plasma)<br>Month 33	10,339,648	3, 542.83	215 ng<br>(breast biopsy; FFPE)<br>Month 5	9,668,240	3,547.94	400 ng<br>(normal tissue; FFPE)	9, 953,971	3,395.04
Pt-025	120 ng<br>(serum)<br>Month 19	5,707,795	882.45	300 ng<br>(skin biopsy; OCT)<br>Month 19	7,562,846	2,455.77	Not available		
Pt-026	20 ng<br>(serum)<br>Month 1	2,474,297<br>Middle output	811.19<br>Middle output	178 ng<br>(breast biopsy; OCT)<br>Month 1	7,405,013	2,660.17	Not available		
Pt-027	39 ng<br>(plasma)<br>Month 1	5,191,168	1,619.44	No tissue available	N/A	N/A	Not available		
Pt-028	55 ng<br>(serum)<br>Month 15	5,307,122	1,686.17	158 ng<br>(skin biopsy; OCT)<br>Month 15	10,561,638	3,420.22	75 ng<br>(normal tissue; FFPE)	9,155,511	3,219.77
Pt-029	60 ng<br>(serum)<br>Month 33	5,694,013	2,010.89	46 ng<br>(pleural fluid; OCT)<br>Month 33	8,999,700	3,228.20	Not available		
Pt-030	68 ng<br>(serum)<br>Month 50	5,368,224	938.61	300 ng<br>(pleural fluid; OCT)<br>Month 50	4,460,607	1,435.68	Not available		
Pt-031	17 ng<br>(plasma)<br>Month 25	5,536,747	1,813.84	211 ng<br>(skin biopsy; FFPE)<br>Month 31	6,982,961	2,447.13	Not available		
Pt-032	23 ng	3,426,829	1,117.79	No tissue available	N/A	N/A	Not available		

	(plasma) Month 8								

Supplementary Table 6. Total number of reads and mean read depth by NGS. The quantity of DNA to prepare the libraries, number of reads and mean read depth are indicated for each sample. The time of collection of the sample is indicated in each case. For Pt-001, we also studied cfDNA plasma collected at month 22. The samples that were collected at the same time point (blood and paired tumor tissue or cells) are highlighted in yellow.
